# Arhgap28 Is a RhoGAP that Inactivates RhoA and Downregulates Stress Fibers

**DOI:** 10.1371/journal.pone.0107036

**Published:** 2014-09-11

**Authors:** Ching-Yan Chloé Yeung, Susan H. Taylor, Richa Garva, David F. Holmes, Leo A. Zeef, Raija Soininen, Raymond P. Boot-Handford, Karl E. Kadler

**Affiliations:** 1 Wellcome Trust Centre for Cell-Matrix Research, University of Manchester, Manchester, United Kingdom; 2 Faculty of Life Sciences, University of Manchester, Manchester, United Kingdom; 3 Department of Dermatology, Oulu Center for Cell-Matrix Research, University of Oulu, Oulu, Finland; University of Birmingham, United Kingdom

## Abstract

The small GTPase RhoA is a major regulator of actin reorganization during the formation of stress fibers; thus identifying molecules that regulate Rho activity is necessary for a complete understanding of the mechanisms that determine cell contractility. Here, we have identified Arhgap28 as a Rho GTPase activating protein (RhoGAP) that switches RhoA to its inactive form. We generated an *Arhgap28*-*LacZ* reporter mouse that revealed gene expression in soft tissues at E12.5, pre-bone structures of the limb at E15.5, and prominent expression restricted mostly to ribs and limb long bones at E18.5 days of development. Expression of recombinant Arhgap28-V5 in human osteosarcoma SaOS-2 cells caused a reduction in the basal level of RhoA activation and disruption of actin stress fibers. Extracellular matrix assembly studies using a 3-dimensional cell culture system showed that *Arhgap28* was upregulated during Rho-dependent assembly of the ECM. Taken together, these observations led to the hypothesis that an Arhgap28 knockout mouse model would show a connective tissue phenotype, perhaps affecting bone. Arhgap28-null mice were viable and appeared normal, suggesting that there could be compensation from other RhoGAPs. Indeed, we showed that expression of *Arhgap6* (a closely related RhoGAP) was upregulated in Arhgap28-null bone tissue. An upregulation in *RhoA* expression was also detected suggesting that Arhgap28 may be able to additionally regulate Rho signaling at a transcriptional level. Microarray analyses revealed that *Col2a1*, *Col9a1*, *Matn3*, and *Comp* that encode extracellular matrix proteins were downregulated in Arhgap28-null bone. Although mutations in these genes cause bone dysplasias no bone phenotype was detected in the Arhgap-28 null mice. Together, these data suggest that the regulation of Rho by RhoGAPs, including Arhgap28, during the assembly and development of mechanically strong tissues is complex and may involve multiple RhoGAPs.

## Introduction

The actin cytoskeleton is fundamental to a wide range of cellular functions including cellular contractility, stiffness sensing, tissue formation, cell migration and cell polarity but the molecular mechanisms are complex and not fully understood. Members of the family of Rho guanosine triphosphatases (GTPases) are major regulators of the assembly of actin-based stress fibers along with mammalian diaphanous 1 (mDia) and Rho-associated kinase (ROCK) [1]–[3]. ROCK positively drives the assembly of contractile actin stress fibers by directly phosphorylating the myosin light chain (MLC), and also by inactivating MLC phosphatase [2], [4]. Dynamic reorganization of the actin cytoskeleton into stress fibers is essential for fibronectin assembly and is regulated by signaling from Rho GTPases [5], [6]. Actomyosin contractility is required for the translocation of fibronectin-bound integrins in specialized cell-matrix adhesions along actin stress fibers, a process that is believed to stretch folded fibronectin dimers to facilitate their assembly [7]–[9]. The mechanism for stress fiber-mediated ECM assembly is mechano-sensitive (via cell-matrix adhesions) and tightly regulated. For examples, disruption to actin polymerization or loss of tension caused the misalignment of collagen fibrils in a newly synthesized tendon ECM [10], [11], and targeted ROCK overexpression in the epidermis led to increased collagen deposition and ECM stiffness [12]. While it is well known that Rho GTPases regulate actin stress fiber assembly, how they are regulated during tissue morphogenesis is less well understood.

Although Rho is a GTPase its rate of GTP hydrolysis slow. Efficient hydrolysis of GTP requires a Rho GTPase activating protein (RhoGAP), which accelerates the hydrolytic activity up to 105-fold [13]. There are over 70 genes encoding proteins that contain a RhoGAP domain [14]. This multitude of RhoGAPs is thought to ensure signaling specificity, for example, via tissue-specific expression, specificity for a single GTPase or signaling pathway, or that some RhoGAPs act as scaffold proteins or effectors for crosstalk between Rho GTPases and other signaling pathways (reviewed by [14]).

We show here that *Arhgap28* is differentially regulated during mouse embryonic development. The functions of Arhgap28 have not been reported but its differential expression has been listed in a variety of cDNA microarray studies, as summarized in Table S1. Based on amino acid sequence similarities Arhgap28 is closely related to Arhgap6, Arhgap11a, Arhgap11b, Arhgap18, Arhgap40, DLC1 (Arhgap7), DLC2 (Arhgap27) and DLC3 (Arhgap38). Some of these RhoGAPs have been shown to regulate actin reorganization. Knockout of DLC1 is embryonic lethal at E10.5 of mouse development and examination of fibroblasts isolated from E9.5 mouse embryos revealed disrupted stress fibers and focal adhesions [15]. Mice lacking functional Arhgap6 protein are phenotypically normal, despite the fact that Arhgap6 is a RhoGAP for RhoA and causes the loss of actin stress fibers in cultured cells [16]. Arhgap18 also has specificity for RhoA and disrupts actin stress fibers, where knockdown of Arhgap18 can enhance stress fiber formation [17]. On the basis of these studies, we hypothesized that Arhgap28 regulates actin stress fiber assembly.

## Results

### Sequence alignment predicts that Arhgap28 has a RhoGAP function similar to Arhgap6 and Arhgap18

**Figure 1:**
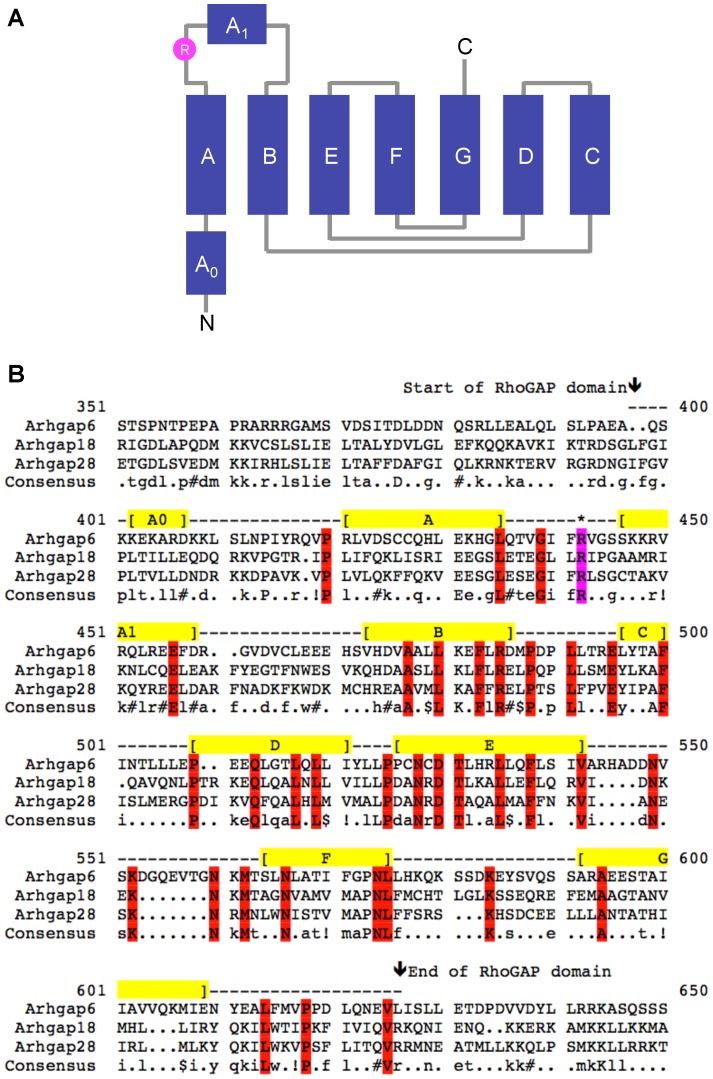
Sequence alignment of the RhoGAP domain of Arhgap28-related RhoGAPs. **A**. Schematic of the helical segments and loops in the RhoGAP domain. The catalytic arginine residue is highlighted in pink. **B**. The amino acid sequences of Arhgap6, Arhgap18 and Arhgap28 were aligned and the sequences of RhoGAP domain are shown. The helical segments (A0, A, A1, B, C, D, E, F and G) are shown in yellow. Residues conserved in all three RhoGAPs are highlighted in red. The catalytic arginine residue is highlighted in pink.

RhoGAP function is mediated via the RhoGAP domain (represented schematically in Figure 1A), which enhances hydrolysis of GTP by the target Rho GTPase. Alignment of the RhoGAP domains of murine Arhgap6, Arhgap18 and Arhgap28 showed high homology and confirms the presence of a putative catalytic arginine residue (R425) in Arhgap28 (Figure 1B), suggesting that Arhgap28 has RhoGAP function. No other conserved domains were identified.

### Expression of Arhgap28-V5 inhibits RhoA activation and actin stress fiber assembly

**Figure 2:**
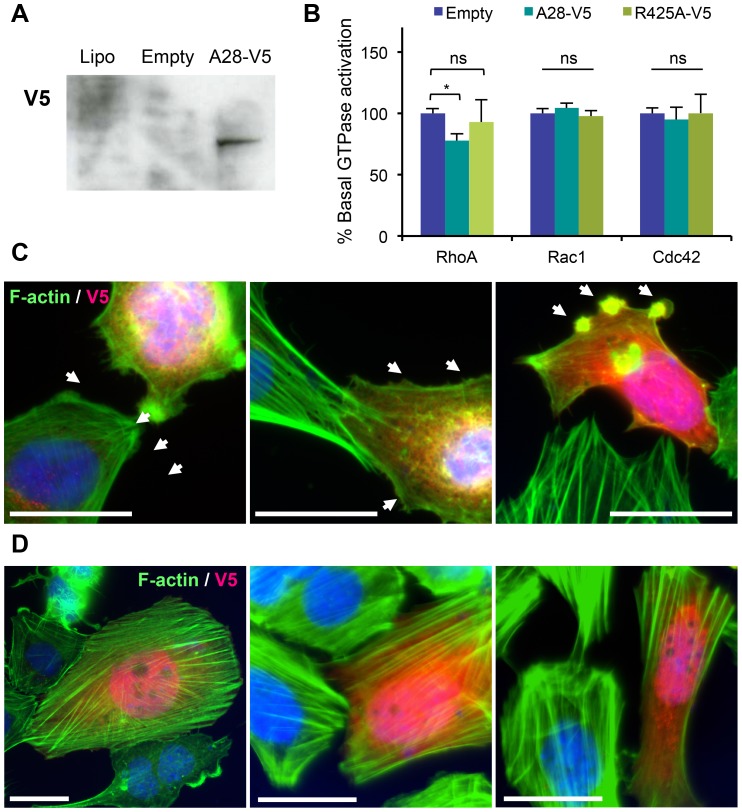
Arhgap28-V5 inhibits RhoA activation and stress fiber formation in SaOS-2 cells. SaOS-2 cells were transfected with empty vector or Arhgap28-V5. **A**. The expression of Arhgap28-V5 was confirmed by western blotting using an antibody to V5. **B**. Effect of Arhgap28-V5 expression on the basal activity of RhoA (n = 5), Rac1 (n = 3) and Cdc42 (n = 3). Bars show SEM. * indicates significant difference found, *p*<0.05. **C**–**D**. F-actin in cells expressing Arhgap28-V5 (**C**) and Arhgap28_R425A_-V5 (**D**) was visualized by fluorescence microscopy using anti-V5 antibodies and Atto 488-conjugated phalloidin (representative images from 3 independent transfections). Arrows point to membrane ruffling and F-actin protrusions. Bars  = 25 µm.

To examine if Arhgap28 can regulate RhoA signaling and actin stress fiber formation, we created a V5-tagged *Arhgap28* expression clone (Arhgap28-V5; see Figure S1 for details) and transfected SaOS-2 cells. SaOS-2 cells were selected because they form prominent stress fibers when cultured on plastic or glass. Expression of Arhgap28-V5 was confirmed in transiently transfected cells by western blotting using an anti-V5 antibody (Figure 2A). Active RhoA was examined by a Rhotekin-GST pull-down assay to precipitate GTP-bound Rho. Expression of Arhgap28-V5 caused a reduction in active RhoA compared to cells treated with the transfection reagent only or when transfected with the empty vector (data not shown). In further experiments we performed quantitative ELISA assays for active RhoA, Rac1 and Cdc42 in the presence and absence of expressed Arhgap28-V5 and a mutant Arhgap28_R425A_-V5 (R425A-V5) with control samples transfected with empty vector only. Note that the R425A-V5 construct lacks the putative catalytic arginine residue in the GAP domain (see Figure 1). We consistently observed reduction in the amounts of active RhoA (but not Rac1 and Cdc42) in the presence of Arhgap28-V5. The amounts of active RhoA remaining ranged from 62–78% of basal levels of active RhoA observed in controls. The results of the experiment that showed reduction to 78% is shown in Figure 2B; the expression of Arhgap28-V5 but not the R425A-V5 resulted in a significant reduction in basal levels of active RhoA with no significant effect on basal Rac1 and Cdc42 activity. The variability in the results between experiments was most probably because of differences in transfection efficiency between experiments. In separate experiments we prepared stably transfected SaOS-2 cells, expressing Arhgap28-V5. However, for reasons that were unclear to us, the expression levels were very low. Overall, the experiments showed that Arhgap28-V5 was effective at reducing the levels of RhoA.

In further experiments, actin stress fibers were examined by staining with phalloidin and immunofluorescence using the anti-V5 antibody; cells without staining for V5 exhibited prominent stress fibers (Figure 2C). In all cells expressing Arhgap28-V5, as identified by staining with V5 antibodies, disrupted actin stress fibers were observed. Multiple actin microspikes and membrane ruffles were also observed on the edge of these cells (Figure 2C, arrows). To test if these morphological changes were attributed to the GTP hydrolysis activity mediated by Arhgap28, cells were transfected with R425A-V5. All cells that stained positively for V5 contained prominent stress fibers (Figure 2D). These expression studies indicated that Arhgap28 is a RhoGAP for RhoA and its expression negatively regulates stress fibers.

### *Arhgap28* has a restricted expression pattern through embryonic development

**Figure 3:**
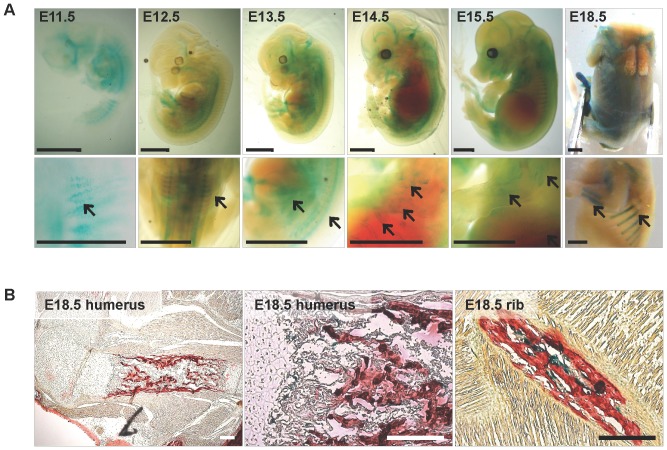
Spatial and temporal expression of *Arhgap28* in *Arhgap28^gt^* reporter mice. Embryos from time-mated heterozygous *Arhgap28^gt^* mice were stained with X-gal and genotyped. **A**. Embryos homozygous for the *Arhgap28^gt^* allele were imaged. Wild type embryos acted as controls (not shown). Bars  = 2 mm. **B**. Homozygous *Arhgap28^gt^* embryos from E18.5 litters were processed for wax embedding and sagittal sections were counterstained with Alizarin red S to stain for calcified matrix. Areas of where X-gal-positive cells are shown. Cells stained positive with X-gal are blue green and red shows tissues stained with Alizarin red S. Bars  = 200 µm.

We studied the spatial and temporal activation of *Arhgap28* during embryonic development using an *Arhgap28* gene trap (*Arhgap28^gt^*) mouse. The gene trap cassette does not disrupt the expression of the wild type *Arhgap28* transcript and so the animals develop normally (see Figure S2 for details). In this mouse, the endogenous promoter of *Arhgap28* drives the expression of β-galactosidase, which can be localized by X-gal staining. We examined the expression of β-galactosidase in E11.5 to E18.5 embryos. At all time points examined, β-galactosidase activity had a restricted spatial pattern (Figure 3A). At E11.5 and E12.5, staining was localized to the dorsal region in what appears to contain somatic cells (Figure 3A arrows in lower panels). At E13.5 to E15.5 the staining in the body of the embryos spread to the limbs and regions where the ribs are formed. At E18.5, staining could be clearly seen in limb bones and in the dorsal portions of the ribs. E18.5 embryos were sectioned and counter stained with Alizarin red. The results showed that cells staining positively for β-galactosidase activity were localized to the calcified portions of long bones and ribs (Figure 3B). These data suggested to us that Arhgap28 has a role in regulating Rho in the initial stages (from E12.5) of ECM assembly throughout the embryo and becomes restricted to boney tissues at late stages of embryonic development, in the mouse.

### *Arhgap28* and related RhoGAPs are upregulated during Rho-mediated tissue stiffening *in vitro*

**Figure 4:**
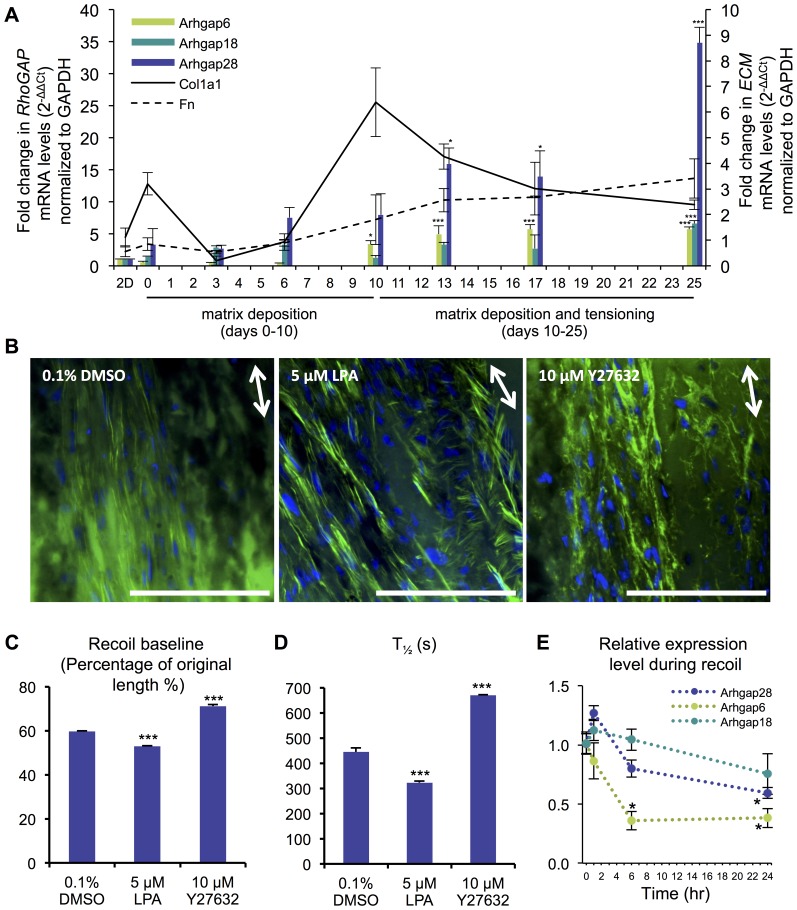
*Arhgap28* is upregulated during ECM assembly and downregulated during Rho-dependent tissue recoil. Primary embryonic chick fibroblasts were seeded into a fibrin gel for the formation of 3D cell cultures containing aligned ECM. **A**. The expression of *Arhgap6*, *Arhgap18*, *Arhgap28, Col1a1*, and *Fn* were quantified by qPCR (n = 3). **B**. 3D tissue constructs were treated with 5 µM LPA or 10 µM Y27632 for 2 hours in serum-free conditions and changes to the actin cytoskeleton were observed by phalloidin staining. Bars  = 50 µm. Arrows indicate the alignment of the tissue. **C**. The recoil baseline, which is the calculated length of the construct at the end of the exponential fit (see Materials and Methods for details). **D**. The half-time, which is the time required to achieve half of the recoil baseline. **E**. The expression of *Arhgap6*, *Arhgap18* and *Arhgap28* were quantified by qPCR during tissue recoil (n = 3). Fold changes in gene expression were normalized to *Gapdh* (2^−ΔΔCt^ values). Bars show SEM. *** and * indicate significant differences found, *p*<0.001 and *p*<0.05, respectively, one way ANOVA.

To further explore the possibility that Arhgap28 is involved in the assembly of ECM, we examined the expression of *Arhgap28* in a 3D cell culture model of tissue assembly in which embryonic fibroblasts deposit and tension a collagen fibril-rich ECM [11]. In this system, fibroblasts are moved from conventional 2D culture to medium containing fibrinogen and thrombin. The formation of a fibrin gel occurs within 5 minutes to produce a loose gel in which the fibroblasts find themselves suspended. During the next ∼10 days in culture, the fibroblasts replace the fibrin with a collagen/fibronectin-rich matrix [18] and subsequently tension this matrix using non-muscle myosin II-derived forces [19]. Quantitative PCR showed that the expression of *Arhgap28* was low in cells cultured on plastic (Figure 4A). The expression increased when the cells were in fibrin gels but was notably upregulated (15-fold) once the tissue constructs had tensioned (day 13; *p*<0.05). The expression levels continued to increase and were 35-fold higher (compared to 2D culture) after 14 days of 3D culture under tension (25 days in total; *p*<0.001; Figure 4A). *Arhgap6* and *Arhgap18* were also upregulated during tissue construct formation but not to the same extent as *Arhgap28* (Figure 4A).

To test if Rho signaling is involved in 3D tissue construct contraction, fully formed tissue constructs (10 days of culture) were treated with lysophosphatidic acid (LPA) to activate Rho and the formation of stress fibers [20], [21] or the ROCK inhibitor, Y27632. As shown in Figure 4B, actin polymerization was dramatically affected by these treatments; LPA induced thicker, more prominent actin stress fibers whereas Y27632 resulted in stress fiber shortening and disassembly.

The 3D tissue constructs recoil when unpinned, which is a process driven by non-muscle myosin II-dependent actomyosin contraction [22]. We made use of this feature to develop an assay of Rho-myosin II-dependent contraction of the tissue constructs. Thus, constructs were incubated with either LPA or Y27632 and the length of the unpinned construct was measured during 30 minutes. Tissue constructs treated with LPA significantly contracted to 53%±0.02 of the original length in 30 minutes, compared to 59.6%±0.04 in DMSO-treated tissue constructs (*p*<0.001; Figure 4C). Y27632 treatment significantly inhibited the contraction of the construct, which contracted to only 71.2%±0.6 of the original length after 30 minutes (*p*<0.001; Figure 4C). The T_1/2_ for recoil was: 444 s±16 for DMSO-treated control tissue constructs, 323 s±7 for LPA treated constructs, and 670 s±3 for Y27632 treated constructs (Figure 4D). In addition, expression of *Arhgap28* and *Arhgap6* was dramatically downregulated at 6 and 24 hours after tissue constructs were unpinned (Figure 4E). Together, these data show that *Arhgap28* is upregulated and Rho signaling is active during contraction of newly synthesized ECM, and the expression of *Arhgap28* is inversely related to the stiffness of the mechanical environment.

### Normal embryonic development of Arhgap28-null mice

To test further the possible role of Arhgap28 in tissue formation, we generated a functional Arhgap28-null mouse by crossing *Arhgap28^gt^* mice with *Cre* transgenic mice. LoxP sites flank exons 7, 8 and 9 of the *Arhgap28^gt^* gene. Therefore, Cre recombinase would be predicted to produce mice harboring an *Arhgap28 del7-9* (*Arhgap28^del^*) allele (Figure S3A). Using PCR readouts with genomic DNA we were able to identify wild type, heterozygous and null mice (Figure S3B). The absence of exons 7–9 causes a frame shift and a smaller transcript that can be detected by RT-PCR using primers that span exons 6 to 11 (Figure S3C). Thus, transcripts from the defective allele would produce a truncated protein lacking the RhoGAP domain. The results showed that heterozygous *Arhgap28^del^* mice were viable and fertile. Litters produced by cross breeding heterozygotes had a close to normal Mendelian distribution of genotypes: 30%±9 wild type; 49%±5 heterozygotes; and 22%±8 homozygotes (±SEM; from a total of 37 pups, in 8 litters). *Arhgap28^del^* embryos (E15.5) were examined histologically and no apparent differences were observed compared to wild type mice (Figure S3D).

### *Arhgap6* and *RhoA* are upregulated in Arhgap28-null bone tissue

**Figure 5:**
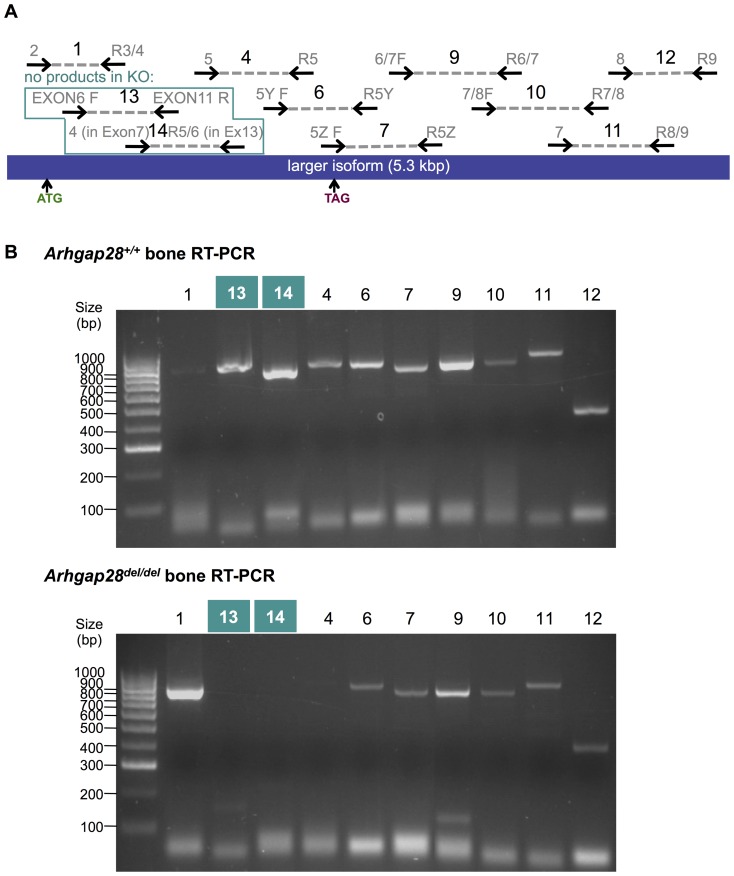
Comparison of the full-length transcripts of wild type and *Arhgap28^del^* alleles. **A**. Overlapping products of PCR reactions 1, 4, 6–7, 9–14 by specific primers (numbers in gray) are sequenced. ATG and TAG indicates the start and stop codons, respectively. Diagram not drawn to scale. **B**. RNA was isolated from the tibia and fibular of P0 mice, and the 10 overlapping RT-PCRs were performed and visualized by gel electrophoresis. Products were subsequently purified and confirmed by DNA sequencing. Reactions in the green boxes will not produce a product with cDNA from *Arhgap28^del^* (see Figure S4).

To confirm the loss of Arhgap28 in bone tissues, RNA was isolated from the tibia and fibula of P0 wild type and *Arhgap28^del^* mice, and overlapping PCRs were performed (Figure 5A). Full-length *Arhgap28* was detected in wild type bone tissues and overlapping PCRs confirmed that exons 7–9 were absent in the *Arhgap28* transcript of *Arhgap28^del^* bone tissues (Figure 5B). The products of these reactions were sequenced to confirm that the transcript expressed in *Arhgap28^del^* bone encoded a 367 amino acid-long protein lacking a functional RhoGAP domain (Figure S4).

**Figure 6:**
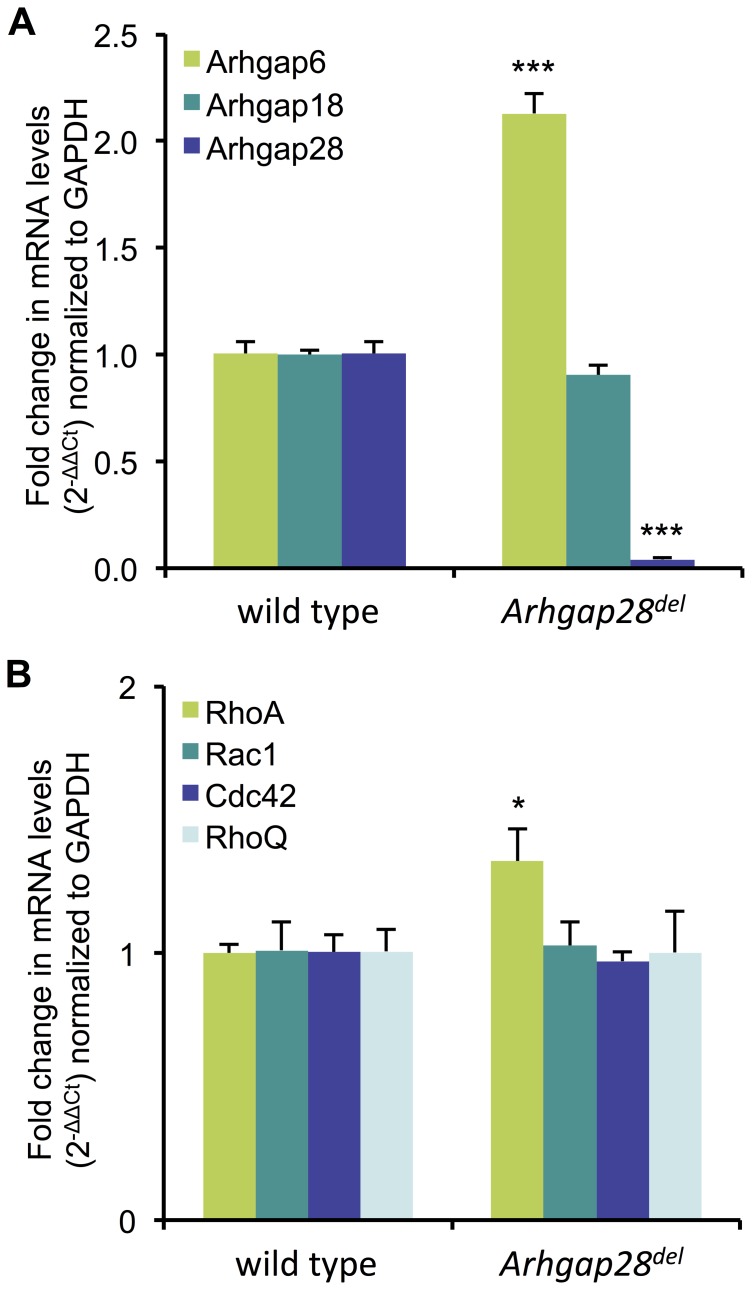
Expression of Rho GAPs and Rho GTPases in *Arhgap28^del^* bone tissues. RNA was isolated from bone tissues (from tibia and fibula) of P0 wild type and *Arhgap28^del^* mice. **A** and **B**. The expression of genes encoding Arhgap28 and related RhoGAPs, Arhgap6 and Arhgap18 (panel **A**); and Rho GTPases, RhoA, Rac1, Cdc42 and RhoQ (panel **B**) was quantified by qPCR (n = 3). Fold changes in gene expression normalized to *Gapdh* (2^−ΔΔCt^ values). Bars show SEM. *** and * indicate significant differences found when compared to wild type, *p*<0.001 and *p*<0.05, respectively.

Due to the lack of an obvious phenotype in heterozygous or null mice, we investigated the possibility that other RhoGAPs (e.g. Arhgap6 and Arhgap18) were compensating for the absence of Arhgap28. Quantitative PCR showed that the truncated *Arhgap28^del^* transcript was detectable at low levels in tissues from *Arhgap28^del^* mice, which suggests that this truncated transcript (lacking the sequences encoding the RhoGAP domain) is at least partially stable. However, it was clear that the mutant transcript was at significantly lower levels compared to the expression of the *Arhgap28* transcript in wild type mice (*p*<0.001; Figure 6A) presumably due to nonsense mediated mRNA decay. We also detected a significant 2-fold upregulation of *Arhgap6* expression in *Arhgap28^del^* tissues (*p*<0.001). Noteworthy, no significant differences were found in the expression of *Arhgap18* in *Arhgap28^del^* tissues (Figure 6A). The data show that in tissues devoid of functional Arhgap28, there is compensatory upregulation of *Arhgap6*. We have previously shown that the two RhoGAPs were upregulated during ECM assembly and tensioning and were downregulated in response to lack of tension, which indicate that the Arhgap6-Arhgap28 pair might co-regulate the same Rho signaling pathway for actin reorganization. We also examined the expression of Rho GTPases. A small but significant upregulation in *RhoA* expression, but not in expression of *Rac1*, *Cdc42* or *RhoQ*, was found in *Arhgap28^del^* bone tissues compared to wild type (*p*<0.05; Figure 6B). Again, these data were indicative of a role of Arhgap28 in RhoA regulation.

### Microarray comparison of gene expression between bone tissues of wild type and *Arhgap28^del^* mice

While *Arhgap6* expression was upregulated in *Arhgap28^del^* bone tissues there was also a significant increase in *RhoA* gene expression, which suggests that Rho signaling might still be altered in *Arhgap28^del^* tissues despite *Arhgap6* compensation. To determine if global changes in gene expression occurred, microarray analyses were performed in which gene expression from bone tissues (tibia and fibula) of wild type and *Arhgap28^del^* mice were compared. The integrity of the RNA samples and the microarray readouts were analyzed (see Figure S5 for details). A total of 45037 probe sets were detected of which 363 of these showed significant differential expression of ≥±2-fold difference (*q*<0.05). A heat map generated by hierarchical clustering of all the probe sets, based on similarities in the expression level and the expression profile is shown in Figure S5D.

As a cautionary note, three probe sets detected *Arhgap28^del^* expression in bone from knockout animals (see Table 1). This was not unexpected because a 3′ microarray was used and the mutant *Arhgap28^del^* transcript contains the endogenous 3′-end of the *Arhgap28* transcript (see Figure 5). In contrast to the qPCR analyses performed previously, readouts for *Arhgap6* did not show a fold change of ≥±2 in *Arhgap28^del^* bone (Table 1). Expression of *Arhgap18* and Rho GTPases (*RhoA*, *Rac1*, *Cdc42* and *RhoQ*) also showed no differential expression in *Arhgap28^del^* bone. The discrepancy between the microarray and the qPCR analyses could be because qPCR is a more quantitative and sensitive method of detecting gene expression. Interestingly, the actin genes detected by this microarray study, *Acta1* and *Actc1*, were both significantly downregulated (∼3-fold) in the bone tissue of *Arhgap28^del^* mice (*q*<0.05; Table 1). The collagen genes expressed in bone tissues, *Col1a1*, *Col1a2* and *Col10a1*, were not significantly different in Arhgap28-null bones.

**Table 1:** Expression of genes of interest in *Arhgap28^del^* bone tissues.

Gene	Gene symbol	wild type	*Arhgap28^del^*	Fold change	*q* value
**RhoGAP genes**					
Arhgap28	*Arhgap28*	271.7	110.3	−2.5	0.037
		82.5	76.5	−1.1	0.345
		67.1	64.2	−1	0.466
Arhgap6	*Arhgap6*	62.2	62.1	−1	0.656
		271.4	275.8	1	0.607
		20.2	24.7	1.2	0.257
Arhgap18	*Arhgap18*	488.6	397.5	−1.2	0.106
		82.2	73.2	−1.1	0.356
Deleted in liver cancer 1	*DLC1*	48.6	52.3	1.1	0.413
**Rho GTPases genes**					
RhoA	*RhoA*	5112.5	5060.3	−1	0.627
		4777.4	4752.9	−1	0.643
		12.9	16.3	1.3	0.099
Rac1	*Rac1*	3672.1	3349.2	−1.1	0.254
		3591.8	3611.5	1	0.645
		14.5	18.0	1.2	0.085
Cdc42	*Cdc42*	239.8	190.0	−1.3	0.197
		4229.0	3745.1	−1.1	0.191
		3386.3	3059.5	−1.1	0.352
		8834.9	8114.0	−1.1	0.27
RhoQ	*RhoQ*	2126.4	2001.0	−1.1	0.52
**actin genes**					
alpha-skeletal actin	*Acta1*	334.1	94.0	−3.6	0.015
alpha-cardiac-actin	*Actc1*	687.1	223.8	−3.1	0.046
**bone collagen genes**					
collagen, type I, alpha 1	*Col1a1*	10217.8	10997.6	1.1	0.413
		10473.8	11427.3	1.1	0.528
collagen, type I, alpha 2	*Coll1a2*	321.6	767.0	2.4	0.163
		23205.8	24471.2	1.1	0.422
		19122.7	1024.8	1.1	0.444
collagen, type X, alpha 1	*Col10a1*	1917.6	1024.8	−1.9	0.056

Comparison of gene expression in wild type and *Arhgap28^del^* bone tissues. Mean intensities from hybridization of triplicate samples to probe set(s) for the genes listed.

DAVID online tool was used to perform gene ontology analyses on probe sets showing a differential expression ≥±2-fold. The analysis showed that 57 probe sets detected genes that were downregulated and 306 probe sets detected genes that were upregulated, in the mutant samples. DAVID identified 10 functional annotation clusters in the genes that were downregulated. The top 3 annotation clusters and the genes that are over-represented in these clusters are listed in Table 2. All three clusters contained genes indicative of a cartilage ECM, for example, *Col2a1*, *Col9a1*, *Hapln1* (hyaluronan and proteoglycan link protein 1), *Matn1* (matrillin 1) and *Matn3*, and some genes that are linked to negatively regulating bone mass, *Agtr2* (angiotensin II receptor, type 2) and *Dlk1*, which encodes a transmembrane protein called delta-like 1 homolog. These data suggest that a bone phenotype could still be expected upon closer analysis.

**Table 2:** Top 3 annotation clusters of genes downregulated in *Arhgap28^del^* bone.

Cluster components^A^	*p* value^B^	Matched genes^C^	Gene names (if annotated) of corresponding probe IDs in the GO list^D^
***Annotation cluster 1, Enrichment score 6.72***			
SP_PIR_KEYWORDS secreted	4.13E–11	21	*Egfl6, Hapln1, Mia1, Sfrp4, Ccl6, Col2a1, Lect1, Ptn, Matn3, Ecrg4, Matn3, Hapln1, Ccl12, Comp, C1qtnf3, Angptl1, Hapln1, Nts, Mfap5, Hapln1, Epyc, Ecrg4, Mmrn1, Cma1, Col9a1, Cxcl14*
GO: 0005576∼ extracellular region	6.53E–10	22	*Egfl6, Hapln1, Mia1, Sfrp4, Ccl6, Col2a1, Lect1, Ptn, Matn3, Ecrg4, Hapln1, Matn3, Ccl12, Comp, C1qtnf3, Angptl1, Hapln1, Nts, Mfap5, Matn1, Hapln1, Epyc, Ecrg4, Mmrn1, Cma1, Col9a1, Cxcl14*
SP_PIR_KEYWORDS signal	1.06E–07	24	*Egfl6, Mia1, Sfrp4, Ccl6, Col2a1, Snorc, Dlk1, Ptn, Matn3, Ecrg4, Matn3, Ccl12, Comp, C1qtnf3, Cd300ld, Angptl1, Nts, Mfap5, Matn1, Cpa3, Epyc, Ecrg4, Mmrn1, Cma1, Col9a1, Cxcl14*
GO: 0044421∼ extracellular region part	1.57E–07	14	*Hapln1, Hapln1, Angptl1, Egfl6, Dlk1, Mfap5, Matn1, Col2a1, Hapln1, Epyc, Ptn, Matn3, Hapln1, Matn3, Col9a1, Ccl12, Cxcl14, Comp*
UP_SEQ_FEATURE signal peptide	7.64E–07	24	*Egfl6, Mia1, Sfrp4, Ccl6, Col2a1, Snorc, Dlk1, Ptn, Matn3, Ecrg4, Matn3, Ccl12, Comp, C1qtnf3, Cd300ld, Angptl1, Nts, Mfap5, Matn1, Cpa3, Epyc, Ecrg4, Mmrn1, Cma1, Col9a1, Cxcl14*
UP_SEQ_FEATURE disufide bond	1.86E–06	21	*Egfl6, Hapln1, Mia1, Sfrp4, Ccl6, Lect1, Dlk1, Ptn, Matn3, Matn3, Hapln1, Ccl12, Comp, Agtr2, Cd300ld, Angptl1, Hapln1, Matn1, Cpa3, Hapln1, Epyc, Mmrn1, Cma1, Col9a1, Cxcl14*
***Annotation cluster 2, Enrichment score 3.78***			
GO: 0005578∼ proteinaceous ECM	1.48E–07	10	*Egfl6, Hapln1, Hapln1, Mfap5, Matn1, Col2a1, Hapln1, Epyc, Ptn, Matn3, Matn4, Hapln1, Col9a1, Comp*
GO: 0031012∼ extracellular matrix	2.07E–07	10	*Egfl6, Hapln1, Hapln1, Mfap5, Matn1, Col2a1, Hapln1, Epyc, Ptn, Matn3, Matn4, Hapln1, Col9a1, Comp*
SP_PIR_KEYWORDS extracellular matrix	1.48E–05	7	*Hapln1, Hapln1, Egfl6, Hapln1, Mfap5, Col9a1, Comp, Col2a1, Hapln1, Epyc*
GO: 0007155∼ cell adhesion	7.13E–03	6	*Hapln1, Hapln1, Egfl6, Mia1, Hapln1, Col9a1, Comp, Col2a1, Hapln1*
GO: 0022610∼ biological adhesion	7.19E–03	6	*Hapln1, Hapln1, Egfl6, Mia1, Hapln1, Col9a1, Comp, Col2a1, Hapln1*
***Annotation cluster 3, Enrichment score 2.24***			
SP_PIR_KEYWORDS egf-like domain	2.32E–04	6	*Egfl6, Matn3, Mmrn1, Comp, Matn1, Dlk1, Matn3*

Annotation cluster analysis of probe sets detecting a significant fold change greater than −2 from wild type to *Arhgap28^del^* produced 10 clusters. The top 3 annotation clusters with the highest enrichment score are listed here. **A**. The top gene ontology components of the cluster. **B**. The statistical significance of this grouping where the lower the score the more unlikely this clustering is due to chance. **C**. The number of probe sets that recognize genes contributing to the GO term. **D**. List of gene names of the Affymetrix Mouse Genome 430 2.0 array probe IDs.

For genes that were upregulated in Arhgap28-null bone tissue, 65 annotation clusters were identified and the top 3 clusters are listed in Table 3. The genes that were over-represented in the first cluster were genes involved in targeting proteins for ubiquitination. For example, this cluster contained 13 E3 ubiquitin protein ligases, including *March3* (membrane-associated ring finger (C3HC4) 3), *March5*, *Mybp2* (MYC-binding protein 2); 3 peptidases; and a gene, *Psmd14*, which encodes a regulatory subunit of 26S proteasome (see Table 3 for full list of genes). The second cluster contained genes that promote actin polymerization, including *Pafah1b1* (platelet-activating factor acetyl-hydrolase 1b), *Tmod1* (tropomodulin 1) and *Diap3* (also known as *mDia2*); actin nucleation (*Spire1*); and genes involved in linking the actin cytoskeleton to the plasma membrane including *Utrn* (utrophin) and *Spna1* (spectrin). The third cluster of over-represented genes that were upregulated in *Arhgap28^del^* bone tissue contained zinc finger proteins (*Rbm5*, *Neil3* and *Fus*), which bind DNA or RNA. To summarize, the GO analyses showed that loss of Arhgap28-mediated RhoA signaling causes: (i) down-regulation of cartilage ECM genes; (ii) up-regulation in genes involved in targeting proteins for degradation by ubiquitination; and (iii) up-regulation of genes involved in anchorage of the actin cytoskeleton to the plasma membrane. Whether or not Arhgap28 regulation of Rho signaling is involved functionally in cartilage homeostasis, protein degradation of anchorage of actin to the plasma membrane will require further investigation. However, the results presented in Figure 2 are indicative of a role for Arhgap28 in actin stress fiber polymerization at the plasma membrane.

**Table 3:** Top 3 annotation clusters of genes upregulated in *Arhgap28^del^* bone.

Cluster components^A^	*p* value^B^	Matched genes^C^	Gene names (if annotated) of corresponding probe IDs in the GO list^D^
***Annotation cluster 1, Enrichment score 5.02***			
SP_PIR_KEYWORDS ubl conjugation pathway	1.47E–06	19	*March3, Cblb, Herc4, Mycbp2, Ranbp2, March3, Rad18, Fbxl7, Mkrn1, Spopl, Usp7, March5, Phr1, Hip1, Fbxo30, Hace1, March3, Ube2o, Mycbp2, Dtl, Huwe1, Mkrn1, Psmd14, Usp25, Herc1*
GO: 0030163∼ protein catabolic process	1.64E–06	21	*March3, Cblb, Herc4, Mycbp2, Ranbp2, March3, Rad18, Fbxl7, Mkrn1, Spopl, Usp7, March5, Phr1, Hip1, Fbxo30, Usp32, Hace1, March3, Ybey, Usp32, Btrc, Ube2o, Mycbp2, Dtl, Ttll3, Huwe1, Mkrn1, Psmd14, Usp25, Herc1*
GO: 0019941∼ modification-dependent protein catabolic process	1.72E–06	20	*March3, Cblb, Herc4, Mycbp2, Ranbp2, March3, Rad18, Fbxl7, Mkrn1, Spopl, Usp7, March5, Phr1, Hip1, Fbxo30, Usp32, Hace1, March3, Ybey, Usp32, Ube2o, Mycbp2, Dtl, Ttll3, Huwe1, Mkrn1, Psmd14, Usp25, Herc1*
GO: 0009057∼ macromolecule catabolic process	5.25E–06	22	*March3, Cblb, Herc4, Mycbp2, Ranbp2, March3, Rad18, Fbxl7, Mkrn1, Spopl, Usp7, March5, Phr1, Pan3, Hip1, Fbxo30, Usp32, Hace1, March3, Ybey, Usp32, Btrc, Ube2o, Mycbp2, Dtl, Ttll3, Huwe1, Mkrn1, Psmd14, Usp25, Herc1*
SP_PIR_KEYWORDS ligase	6.21E–06	11	*Hace1, March3, Cblb, March3, Herc4, Mycbp2, Btrc, March3, Rad18, Ube2o, Mkrn1, Mycbp2, Huwe1, Mkrn1, March5, Phr1, Hip1*
GO: 0006508∼ proteolysis	2.91E–03	22	*Cblb, March3, Herc4, Mycbp2, Ranbp2, March3, Rad18, Fbxl7, Mkrn1, Spopl, Usp7, March5, Phr1, Hip1, Fbxo30, Usp32, Hace1, March3, Ybey, Usp32, Ube2o, Mycbp2, Dtl, Ttll3, Huwe1, Mkrn1, Psmd14, Kel, Metap2, Usp25, Herc1*
***Annotation cluster 2, Enrichment score 3.38***			
GO: 0003779∼ actin binding	1.03E–04	13	*Myo1D, Spna1, Myo1D, Epb4.1, Spna1, Spire1, Spire1, Utrn, Fhdc1, Trpm7, Tmod1, Mtss1, Epb4.9, Epb4.1, Slc4a1, Epb4.1, Diap3, Add1, Fhdc1*
GO: 0030036∼ actin cytoskeleton organization	3.71E–04	9	*Myo1D, Pafah1b1, Trpm7, Rictor, Fhdc1, Tmod1, Mtss1, Epb4.1, Sorbs1, Epb4.1, Sorbs1, Diap3, Fhdc1*
GO: 0007010∼ cytoskeleton organization	7.02E–04	12	*Myo1D, Pafah1b1, Stradb Trpm7, Rictor, Fhdc1, Tmod1, Mtss1, Epb4.9, Epb4.1, Sorbs1, Epb4.1, Sorbs1, Diap3, Cenpe, Fhdc1*
GO: 0007155∼ cell adhesion	8.28E–04	14	*Myo1D, Spna1, Myo1D, Epb4.1, Pafah1b1, Spna1, Spire1, Spire1, Utrn, Fhdc1, Trpm7, Tmod1, Mtss1, Epb4.9, Epb4.1, Slc4a1, Epb4.1, Diap3, Fhdc1, Add1*
***Annotation cluster 3, Enrichment score 3.15***			
UP_SEQ_FEATURE zinc finger region: RanBP2-type	1.09E–02	3	*Rbm5, Neil3, Fus*

Annotation cluster analysis of probe sets detecting a significant fold change greater than 2 from wild type to *Arhgap28^del/del^* produced 65 clusters. The top 3 annotation clusters with the highest enrichment score are listed here. **A**. The top gene ontology components of the cluster. **B**. The statistical significance of this grouping where the lower the score the more unlikely this clustering is due to chance. C. The number of probe sets that recognize genes contributing to the GO term. **D**. List of gene names of the Affymetrix Mouse Genome 430 2.0 array probe IDs.

### Analyses of bone length in mature mice show no evidence of bone dysplasia

**Figure 7:**
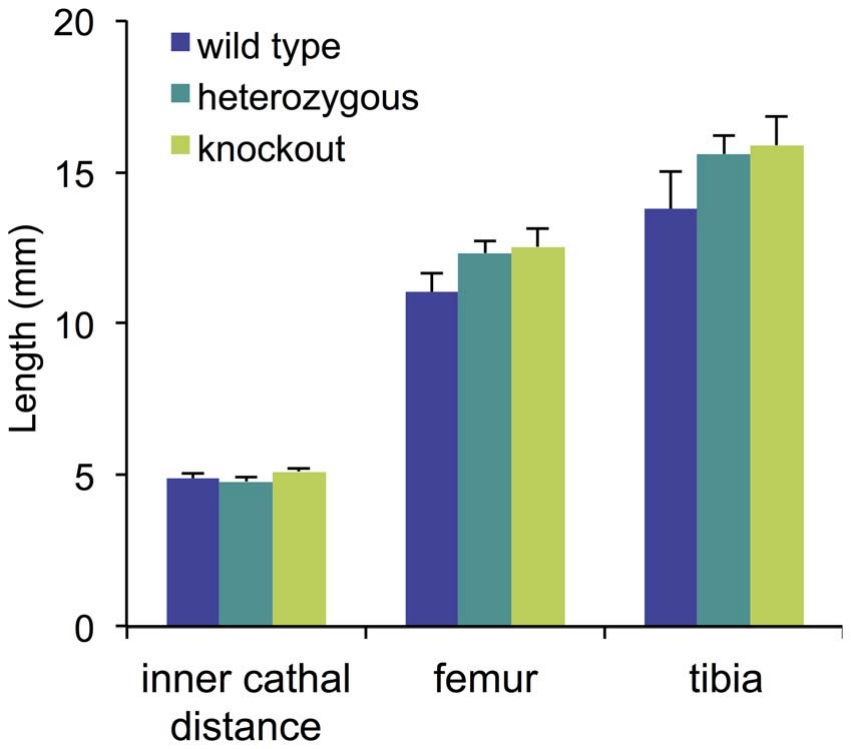
Analysis of bone length of the *Arhgap28^del^* mouse. Length of tibia and femur measured from X-ray images of 10 week-old wild type (n = 5), heterozygous (n = 9) and Arhgap28-null (n = 5) mice.

The results of the GO analyses of Arhgap28-null bone tissues showing down-regulation of genes encoding cartilage ECM molecules and the fact that *Arhgap28* expression was localized to pre-bone structures in embryos, prompted us to explore the possibility of a bone phenotype in the knockout mice, especially because mutations in *Col2a1*, *Col9a1*, *Matn1* and *Matn3* are associated with bone dysplasia (for reviews see [23]–[25]). Thus, we examined the skeletons of mice by X-ray at 10 weeks of age, when the skeletal growth has reached maturity. Skeletal analyses revealed no significant differences in the lengths of femur and tibia between wild type, heterozygotes and *Arhgap28^del^* mice (Figure 7). As a measure of intramembranous ossification, we measured the intercanthal distance and found no significant differences between wild type, heterozygous and Arhgap28-null animals (Figure 7). Together, these data suggest that although genes associated with bone dysplasia were downregulated in the *Arhgap28^del^* mouse there is no bone length phenotype, presumably because of compensation from Arhgap6.

## Discussion

The mechanical stiffness of musculoskeletal tissues is directly related to the organization of collagen fibrils in the ECM. For examples, the strongest tensile tissues such as tendon and ligament have collagen fibrils arranged in parallel register, which presumably is the best organization to resist uniaxial force; in bone the collagen fibrils provide a template for mineralization. Furthermore, the collagen fibrils are pre-stressed by cells to ensure that tissues can respond directly to applied forces. It is poorly understood how the tissue-specific arrangement and pre-stressing of collagen fibrils is achieved. Tissue stiffening requires cellular contraction via actin stress fibers, which are regulated by Rho GTPases. How these Rho GTPases are regulated during tissue development to ensure that the ECM is at optimal stiffness is unknown.

Here, we show that *Arhgap28* is activated in bone tissues before birth and during the assembly of a stiff ECM. Experiments expressing Arhgap28-V5 suggest that Arhgap28 is a negative regulator of RhoA and actin stress fiber formation. Arhgap28-deficiency does not appear to affect bone development, which is most likely due to functional redundancy between Arhgap28 and a closely related RhoGAP, Arhgap6. It will be important in future work to investigate the mechanisms of how Arhgap28-regulated actin contractility determines stiffening of the ECM and to understand how RhoGAPs crosstalk regulates Rho and actin remodeling within developing musculoskeletal tissues.

Cellular tension is generated by actin stress fibers [26]. In this context, the closely related Arhgap6 and Arhgap18 regulate the formation of actin stress fibers via RhoA [16], [17] and in this study, expression of Arhgap28-V5 caused similar effects. Sustained RhoA activity has inhibitory effects on Rac1- and Cdc42-activated lamellipodia and filopodia formation [27], [28], which helps explain the appearance of actin microspikes and membrane ruffles in Arhgap28-overexpressing cells although there was no detectable activity of Arhgap28 against Rac1 and CDC42 in the assays used here.

Cells respond to stiff extracellular matrices via Rho-activated actin stress fibers [29]–[31] and overactive RhoA signaling is linked to cancer [see 32 for review]. Here, loss of RhoGAPs, such as DLC1, is associated with cancer [33], which suggest that RhoA signaling affects cell fate. Overexpression of *RhoA* and Rho GTPases are linked to cancer and the stability of *RhoA* transcripts in cancer cells has been shown to be a result of altered polyadenylation signals [34], further suggesting why it would be biologically important to have more than one RhoGAP regulating Rho GTPases.

There are a few studies that show that the differentiation of mesenchymal stem cells into an osteogenic lineage can be influenced by Rho/ROCK signaling, for examples see [35]–[37]. Surprisingly, mice expressing a dominant-negative RhoA have a bone sclerotic phenotype, which suggests that lack of RhoA signaling enhances mineralization [38]. These disagreements linking Rho/ROCK and bone development could be due to the need for a balanced signal and crosstalk between Rho/ROCK and growth factors rather an all-or-nothing response. *RhoA* expression was also significantly upregulated in bone tissues of Arhgap28-null mice. *RhoA* expression is activated by transcription factor Myc [39], but Myc was not differentially regulated in the microarray comparison between wild type and *Arhgap28^del^* bone tissues.

Due to the large family of mammalian RhoGAPs, it was predicted that Arhgap28-deficiency might be compensated for by functional redundancy between Arhgap28 and other RhoGAPs. Indeed, absence of Arhgap6 in mice does not cause an overt phenotype, presumably because of the observed compensatory mechanisms [16]. It was surprising in our studies that *Arhgap18* was not also upregulated because both Arhgap6 and Arhgap18 have been shown to negatively regulate RhoA and actin stress fibers. These results reveal the potential of a novel co-regulatory mechanism for RhoA signaling and actin stress fibers by Arhgap6 and Arhgap28. How these RhoGAPs are activated during the patterning of ECM is unknown. Matrix assembly and detection of the ECM occurs at cell-matrix adhesion sites, therefore it is hypothesized that Arhgap28 is activated by signals downstream of cell-matrix adhesions in similar mechanisms described for other RhoGAPs. For examples, upon activation of integrin β1, p190-RhoGAP is activated via tyrosine phosphorylation at the N-terminus by Src [40], and more recently, the identification of protein-protein interaction domains within DLC1 suggest direction interaction between DLC1 and cell-matrix adhesion proteins tensin, talin and focal adhesion kinase [41], [42].

Importantly, although there was no overt phenotype in the *Arhgap28^del^* mice (perhaps due to compensation by Arhgap6) the microarray study revealed downregulation in *Acta1* and *Actc1* genes, which encode isoforms of actin found most abundantly in muscle. Downregulation in both these genes has also been observed in a microarray study of chondrocyte differentiation *in vitro* [43] and downregulation of *Acta1* was observed in bone in response to mechanical loading [44], which suggest of change in the cells of *Arhgap28^del^* bone tissues. Gene ontology analyses also revealed that genes that encode ECM molecules associated with a cartilage tissue (including *Col2a1*, *Col9a1*, *Matn3* and *Comp*) were downregulated in Arhgap28-null bone tissues. Mutations in these genes are associated with bone dysplasia [for reviews, see 23,24,25]. Bone tissues from *Arhgap28^del^* mice also showed an upregulation of genes involved in ubiquitination and actin reorganization compared to wild type. Most of the genes enriched in the top cluster were E3 ubiquitin ligases, which mediate the specificity of the ubiquitination pathway [reviewed by 45]. The target for many of these E3 ligases is unknown. The genes that stood out include *Hace1*, which catalyzes the ubiquitination of Rac1 [46], *Mycbp2*, which can bind Myc [47] but whether or not it is inhibitory or enhancing for RhoA transcription is unknown. The differential regulation of these genes is indicative of a response caused by *Arhgap28*-deficiency, which might be independent of RhoA signaling if Arhgap6 compensates for Arhgap28.

In conclusion, we describe experiments that point to Arhgap28 being a functional negative regulator of RhoA during the formation of actin stress fibers in cells of mesenchymal origin. However, knockout experiments in mice indicated that Arhgap28 is not autonomous but is functionally redundant in the presence of other RhoGAPs including Arhgap6 and possibly Arhgap18. Such redundancy presumably underpins the cell's ability to generate actin stress fibers during tissue formation.

## Materials and Methods

### Ethics

The care and use of all mice in this study was carried out in accordance with UK Home Office regulations, UK Animals (Scientific Procedures) Act of 1986 under the UK Home Office licence (PPL 40/3485). No experimental procedures were performed on live animals. All animals were sacrificed by Schedule 1 cervical dislocation by trained personnel, and all efforts were made to minimize suffering.

### V5-His-tagged murine *Arhgap28* and *Arhgap28_R425A_* expression clones

For the generation of a C-terminus V5-His-fusion *Arhgap28* clone, the sequence encoding V5-His (GKPIPNPLLGLDST-(His)6) was introduced immediately 5′ of the stop codon of the endogenous, larger *Arhgap28* isoform cDNA. The cDNA clone of the short *Mus musculus Arhgap28* isoform (BC066788.1; Source BioScience Geneservice) was digested with EcoRI and BsmBI (New England Biolabs, Hertfordshire, UK) following the manufacturer's instructions to produce a 1123 bp fragment of the 5′ portion of the *Arhgap28* clone. For the preparation of the 3′ portion of endogenous *Arhgap28* transcript, a 1209 bp fragment was amplified from mouse fibroblast cDNA (made with random hexamers) using a forward primer 5′ of a unique endogenous BsmBI restriction recognition site 3′-AAG ATT TGG GTT GAC CGA GAC G-5′ and a reverse primer that created a new BstBI site 3′- AAT TCG AAG GGC TTG ATG ACC C-5′ immediately before the stop codon. iProof High-Fidelity DNA Polymerase (Bio Rad Laboratories) was used following the manufacturer's protocol. The PCR fragment was sequenced and cut with BsmBI and BstBI restriction enzymes (New England Biolabs). *pcDNA6/V5-His C* vector was prepared by digestion with EcoRI and BstBI restriction enzymes (New England Biolabs). The three cut fragments were purified using QIAquick Gel Extraction kit (Qiagen) and ligated using T4 DNA Ligase (New England Biolabs). *Arhgap28_R425A_* clone containing a codon change from CGA to GCA (Genscript) was subcloned into *pcDNA6/V5-His C* between EcoRI and BstBI sites. DNA was purified from each culture using QIAprep Spin Miniprep kit (Qiagen) following the manufacturer's protocol.

### Transient transfections and GTPase activation assays

NIH3T3 fibroblasts were cultured in DMEM, supplemented with 10% (v/v) FCS, 2 mM L-glutamine, 10 000 U/ml penicillin and 10 mg/ml streptomycin. SaOS-2 osteosarcoma cells were cultured in RPIM-1630, supplemented with 10% (v/v) FCS, 2 mM L-glutamine, 10 000 U/ml penicillin and 10 mg/ml streptomycin. Cells were cultured at 37°C, 5% CO_2_. DNA was transfected using Lipofectamine 2000 (Invitrogen) according to manufacturer's protocol. Cells were analyzed 24 hours after transfection by indirect immunofluorescence, active GTPase G-LISA assays or western blotting. Expression of V5-tagged constructs was confirmed by western blotting using a mouse monoclonal anti-V5 epitope antibody (MCA1360 from AbD Serotec). The activities of RhoA, Rac1 or Cdc42 were measured 24 hours after transfection using G-LISA Activation Assays (Cytoskeleton), according to manufacturer's protocol.

### 3D tissue constructs and recoil assays

Primary embryonic chick fibroblasts were isolated from E14 chick metatarsal tendons. For the formation of 3D tissue constructs *in vitro*, cells were seeded into fibrin gels as described previously [11]. To modulate Rho signaling *in vitro* tissue constructs were washed with phosphate buffered saline (PBS) three times and then equilibrated for 30 minutes with serum-free medium and then treated for 30 minutes with final concentration of 5 µM lysophosphatidic acid (LPA; Sigma) in 0.1% (v/v) DMSO or 10 µM Y27632 (ROCK inhibitor; Sigma) in 0.1% (v/v) DMSO, diluted in serum-free medium, all at 37°C, 5% CO_2_ in a humidified environment. For the recoil assay, the tissue constructs were treated for 2 hours and was then cut from one suture to allow contraction to occur. The constructs were imaged using a digital single lens reflex camera at a fixed focal point for 30 minutes at 10-second intervals. The length of the constructs in each image was measured using ImageJ software. The length was converted to a percentage of the original and the means for each experimental group were calculated. The mean values were then fitted to a 3-parameter exponential decay function using SigmaPlot (Systat Software Inc.). One-way ANOVA and a Dunnett's test were used to determine significance differences between the derived T_1/2_ and recoil baseline values compared to control constructs incubated in DMSO.

### RNA and PCRs

RNA was isolated with TRIzol reagent (Invitrogen) and treated with DNase (Promega) following manufacturers' protocols. cDNA was synthesized using TaqMan Reverse Transcriptase reagents (Applied Biosystems) and used for analyses by PCR and qPCR. A list of primers used can be found in Table S2. All PCR products produced from primers were validated and confirmed by DNA sequencing using the BLAST software against the NCBI nucleotide database and aligned with the expected sequence. For qPCR analyses, the 2^-ΔΔCt^ method [48] was used to analyze relative fold changes in gene expression compared to the experimental control group. A two-sample t-test or one-way ANOVA followed by a Dunnett's test were performed to determine significance differences compared to the control sample.

### Immunofluorescence

Cells fixed with freshly prepared 4% (w/v) paraformaldehyde in PBS for 20 minutes at RT, then washed with PBS containing 0.1% (v/v) Tween 20 three times and blocked with 1% (w/v) bovine serum albumin, 0.1% (v/v) Triton X-100 in PBS containing 0.1% Tween 20 for 1 hour. Cells were incubated with primary antibodies, mouse monoclonal anti-V5 epitope or the appropriate control IgGs, diluted in the blocking buffer overnight at 4°C. Cells were washed with PBS containing 0.1% (v/v) Tween 20 for 10 minutes three times and incubated with Alexa Fluor 594-conjugated antibodies (Invitrogen) and Atto 488-conjugated phalloidin (Sigma) diluted with the blocking buffer for 1 hour at room temperature, protected from light. Stained cells were washed with PBS containing 0.1% (v/v) Tween 20 for 10 minutes three times and mounted using Vector Shield containing DAPI (Vector Laboratories). Fluorescent images were taken using a digital camera attached to an Olympus BX51 and captured using MetaVue imaging software (Molecular Devices).

### Reporter and knockout mice

Using homologous recombination in agouti C57BL/6N embryonic stem (ES) cell, the L1L2_Bact_P targeting gene trap cassette (including the genes encoding β-galactosidase and neomycin) was introduced into intron 6 of the Arhgap28 gene by the NIH Knockout Mouse Project (KOMP; CA, USA). In addition, exons 7–9 are flanked by loxP sites. ES cells containing the *Arhgap28* gene trap allele (*Arhgap28^gt^*) were injected into C57BL/6J blastocysts to produce germ line-transmitting chimeras (prepared at the University of Oulu, Finland). Chimeric males were assessed on coat color and mated with wild type C57BL/6J females to produce heterozygous progeny, which were then mated to obtain *Arhgap28^gt^* mice. For the generation of *Arhgap28 deleted exons 7*–*9* (*Arhgap28^del^*) mutants, male *Arhgap28^gt^* mice were mated with females from a deleter *Cre* transgenic mice (a gift from M. Briggs, University of Newcastle, UK) to ablate the loxP-flanked exons 7–9 in all tissues. F1 offspring heterozygous for the knockout allele were then mated to generate knockout (*Arhgap28^del^*) mice.

### Genotyping

Mice were genotyped using genomic DNA extracted from ear punches of adult mice or amniotic sacs of embryos using 200 µg/ml Proteinase K (Invitrogen) in buffer containing 17.6 mM N-lauroyl sarcosine, 100 mM NaCl and 5% (w/v) Chelex 100 resin (Bio Rad Laboratories). Genotypes were determined using specific primer pairs (see Table S2) using BioMix Red PCR reagents (Bioline). The annealing temperature for the wild type/mutant allele PCR was 53°C. The annealing temperature for the *Arhgap28^del^* allele was touch-down from 70–60°C for 10 cycles followed by 20 cycles at 60°C. The annealing temperature for the *Cre* transgene was 50°C.

### Whole mount X-gal stain

For detection of beta-galactosidase expression, E10.5–E15.5 embryos were fixed for 1 hour in 3.7% (w/v) PFA in PBS pH 8 at room temperature. For older embryos the skin was removed before being fixed for 1 hour in 3.7% (w/v) PFA in PBS pH 7.4 at room temperature. Embryos were then washed with PBS containing 0.1% (v/v) Triton X-100 for 15 minutes twice. Each embryos was then stained with 20 ml of freshly prepared X-gal staining solution (1 mM X-gal (Qiagen), 5 mM potassium ferricyanide (K_3_Fe(CN)_6_), 5 mM potassium ferrocyanide (K_4_Fe(CN)_6_), 1 mM MgCl_2_ in PBS containing 0.1% (v/v) Triton X-100) for 24 hours at 37°C. After staining, the embryos were rinsed with PBS and post-fixed in 4% (w/v) PFA in PBS overnight at 4°C. After post-fix, the embryos were either processed for paraffin embedding or dehydrated in 70% (v/v) ethanol for 6 hours and cleared with glycerol (10 ml of each 30, 50 and 80% (v/v) glycerol in 1% (w/v) potassium hydroxide and then 100% glycerol), incubating at 37°C for 2-3 days each.

### Histology

Sagittal sections (6 µm thick) were cut from wax embedded embryos and mounted for haematoxylin and eosin staining. Slides were stained using an automated stainer and cleared into Histo-Clear (Thermo Fisher Scientific). For Alizarin red staining, sections were then washed with distilled water and stained with 2% (w/v) Alizarin red pH 4.2 for 10 minutes, washed with distilled water and dehydrated. Images were captured using a Carl Zeiss Axiocam Colour CCD camera with associated AxioVision software.

### Comparative gene expression microarrays

For the comparison of bone tissues between wild type and *Arhgap28^del^* mice tibia and fibula were dissected from three P0 neonatal litters each from defined breedings. Bones were incubated in 1000 U/ml bacterial collagenase type 4 (Worthington Biochemical Corporation) in 0.25% (w/v) trypsin (Invitrogen) for 25 minutes at 37°C with agitation every 10 minutes. The bones were then removed of any excess muscle and cartilage tissues and washed in PBS. RNA was isolated using a dismembrator as described previously [49]. Integrity and measurement of total RNA was performed using Agilent 2100 Bioanalyzer (Agilent Technologies). RNA was amplified by two-cycle cDNA synthesis, and then labelled cRNA was synthesized and hybridized to Mouse Genome 430 2.0 GeneChip arrays (Affymetrix). Microarray data are available in the ArrayExpress database (www.ebi.ac.uk/arrayexpress) under accession number E-MTAB-2296. Microarray data sets were analyzed by dChip (DNA-Chip) Analyzer to normalize the array readouts [50]. Normalized readouts were analyzed using the Robust Multichip Average method as described by [51]. Principal component analysis (PCA) was employed to confirm that different variables were present as a quality control for the arrays. *p* values for each probe set were generated by Limma t-test and *q* values were subsequently generated by applying false discovery rate correction. Gene ontology analysis was performed on probe sets that have detected fold changes greater than 2 using Database for Annotation, Visualization and Integrated Discovery (DAVID) online tool [52].

### Bone length measurements

X-rays of mice were produced using a Flaxitron x-ray specimen radiograph system (Flaxitron Bioptics) and x-ray film (GE Healthcare). Bone measurements were taken from scanned radiographic images using ImageJ software. One-way ANOVA followed by a Dunnett's test were used to determine significance differences compared to wild type.

## Supporting Information

Figure S1 **Cloning of a V5-tagged** ***Arhgap28*** **overexpression construct. A**. Difference in C-terminal ends of the two major *Arhgap28* isoforms from amino acid residue 679. PCR strategy to determine the sequence of endogenous *Arhgap28* transcript expressed in primary mouse fibroblasts. Overlapping products of PCR reactions 1 to 12 by specific primers (numbers in grey) were sequenced. Reactions in the boxes are unique to either the larger or the smaller isoform. ATG and TAG indicates the start and stop codons, respectively. Diagram not drawn to scale. **B**. RT-PCR products with RNA was isolated from primary mouse fibroblasts. A cDNA clone of the smaller *Arhgap28* isoform was used as control template. **C**. *Arhgap28* was cloned into a pcDNA6 vector where a V5-His6 tag was introduced into the *Arhgap28* sequence immediately 5′ of the stop codon. **D**. RNA was isolated from NIH3T3 fibroblasts transiently transfected with Lipofectamine only (L), empty vector (E) or Arhgap28-V5 clones (#6 or #8). RT-PCRs were performed to detect the expression of house-keeping gene, *GAPDH* (221 bp) or *Arhgap28-V5* using specific primers (329 bp). No RT controls confirmed the absence of plasmid DNA contamination. E. Protein was isolated for western blotting to detect V5-tagged protein expression. A positive signal was detected at ∼90 kDa. The predicted molecular weight of Arhgap28-V5 is 85 kDa. Control lysates were included – Talin-V5 (60 kDa), mock transfection (−) and myristoylated-FAK-V5 (+; 175 kDa).
(TIFF)

Figure S2 ***Arhgap28^gt^*** **mice are normal and express** ***Arhgap28*** **due to unsuccessful gene trapping.** **A**. Schematic showing the genotyping strategy for identifying the presence of the gene trap cassette targeted to the *Arhgap28* gene. **B**. DNA was isolated from wild type, *Arhgap28^+/gt^* and *Arhgap28^gt/gt^* mutant neonatal tail tendons cells to confirm genotypes. DNA from wild type (*+/+*) animals will only produce a 493 bp product whereas DNA from homozygous (*gt/gt*) animals will only produce a 354 bp product and DNA from heterozygous (*+/gt*) animals will produce both bands. **C**. RNA was also isolated and RT-PCR was performed to detect the expression of *Arhgap28* transcript spanning from exon 6 to 11, *Col1a1* and *Gapdh*. **D**. Sagittal sections of wild type (*+/+*), heterozygous (*+/gt*) and homozygous *Arhgap28^gt^*mutant (*gt/gt*) embryos at gestation day E15.5 stained with H&E and close-up of the limbs.
(TIFF)

Figure S3 ***Arhgap28^del^*** **mice express an** ***Arhgap28*** **transcript lacking exons 7 to 9.** **A**. Schematic showing the genotyping strategy for identifying the presence of the gene trap cassette targeted to the Arhgap28 gene and for the detection of *Arhgap28 del7-9* KO allele. **B**. Representative gel image of genotyping PCR products. In the first genotyping PCR which distinguishes between wild type (493 bp) or mutant *Arhgap28* allele (either the *Arhgap28^gt^* or *Arhgap28^del^* allele; 354 bp). The second genotyping PCR tests for the presence of the *Arhgap28^del^* allele, the product of the mutant *Arhgap28* allele after Cre recombinase-mediated DNA excision (400 bp). The third genotyping PCR tests for the presence of the deleter *Cre* transgene (350 bp). **C**. RNA was isolated from wild type, *Arhgap28^+/del^* and *Arhgap28^del/del^* pups and RT-PCR was used to detect expression of wild type *Arhgap28* (634 bp) and *Arhgap28^del^* (338 bp) transcripts spanning from exons 6 to 11. RT-PCRs for *Col1a1* and *Gapdh* was used as loading controls. **D**. Sagittal sections of wild type and homozygous *Arhgap28^del^* embryos at gestation day E15.5 stained with H&E.
(TIFF)

Figure S4 **Translated sequences of** ***Arhgap28*** **transcripts.** Amino acid sequences transcripts of *Arhgap28* from wild type, predicted *Arhgap28^del^*, actual *Arhgap28^del^* bone tissue. Sequence in blue is a result of remainder sequence from Cre-mediated recombination. Sequence in green is the RhoGAP domain.
(TIFF)

Figure S5 **Quality control of microarray comparing wild type and** ***Arhgap28^del^*** **bone tissues.** **A**. Agarose gel showing total RNA isolated from triplicate samples of P0 tibia and fibula tissues from wild type and *Arhgap28^del/del^* mice. **B**. PCA mapping of variability between the array samples. **C**. dChip analysis of microarray data sets. (1) Triplicate samples of RNA from tibia and fibula of P0 *Arhgap28^+/+^* (wt) and *Arhgap28^del/del^* (del) mice indicated by the number at the end of each array name. (2) Median intensity of microarray chip for each triplicate of each experimental group. (3) Present (P) call percentage indicates the percentage of total probe sets detected. (4) Array outlier percentage indicates the percentage of probe sets that have outliers in the average readout profile of each probe within a probe set. (5) Percentage single outlier indicates the percentage of probes that do not have the same intensity pattern of other probes within the probe set. **D**. Gene expression changes between P0 wild type and *Arhgap28^del^* bones. Two-dimensional hierarchical cluster heat map analysis of the microarray readouts for all probe sets. This type of clustering is based on similarities in the expression profiles and expression levels. Hierarchical clustering was performed using Cluster 3.0 software and visualised using Java TreeView.
(TIFF)

Table S1 Summary of studies in which *Arhgap28* is identified as a candidate of interest.
(DOCX)

Table S2 PCR primers.
(DOCX)
