# Supplementary figures and images for: Arhgap28 Is a RhoGAP that Inactivates RhoA and Downregulates Stress Fibers
Source: PLoS One. 2014 Sep 11;9(9):e107036. doi: 10.1371/journal.pone.0107036 (PMC4161385; doi:10.1371/journal.pone.0107036)

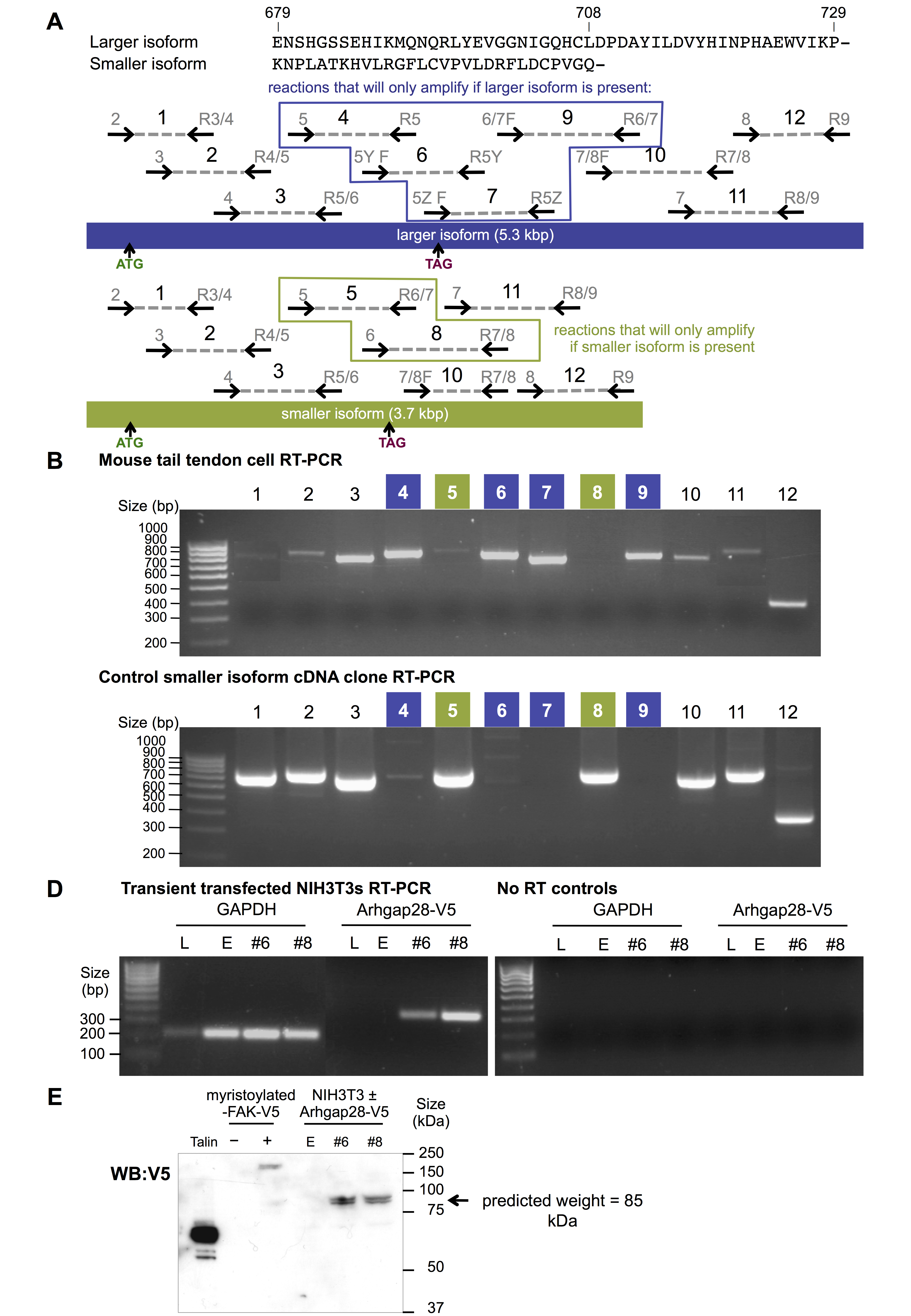

Supplement: Figure S1 — Cloning of a V5-tagged Arhgap28 overexpression construct. A. Difference in C-terminal ends of the two major Arhgap28 isoforms from amino acid residue 679. PCR strategy to determine the sequence of endogenous Arhgap28 transcript expressed in primary mouse fibroblasts. Overlapping products of PCR reactions 1 to 12 by specific primers (numbers in grey) were sequenced. Reactions in the boxes are unique to either the larger or the smaller isoform. ATG and TAG indicates the start and stop codons, respectively. Diagram not drawn to scale. B. RT-PCR products with RNA was isolated from primary mouse fibroblasts. A cDNA clone of the smaller Arhgap28 isoform was used as control template. C. Arhgap28 was cloned into a pcDNA6 vector where a V5-His6 tag was introduced into the Arhgap28 sequence immediately 5′ of the stop codon. D. RNA was isolated from NIH3T3 fibroblasts transiently transfected with Lipofectamine only (L), empty vector (E) or Arhgap28-V5 clones (#6 or #8). RT-PCRs were performed to detect the expression of house-keeping gene, GAPDH (221 bp) or Arhgap28-V5 using specific primers (329 bp). No RT controls confirmed the absence of plasmid DNA contamination. E. Protein was isolated for western blotting to detect V5-tagged protein expression. A positive signal was detected at ∼90 kDa. The predicted molecular weight of Arhgap28-V5 is 85 kDa. Control lysates were included – Talin-V5 (60 kDa), mock transfection (−) and myristoylated-FAK-V5 (+; 175 kDa). (TIFF) [file pone.0107036.s001.tiff]

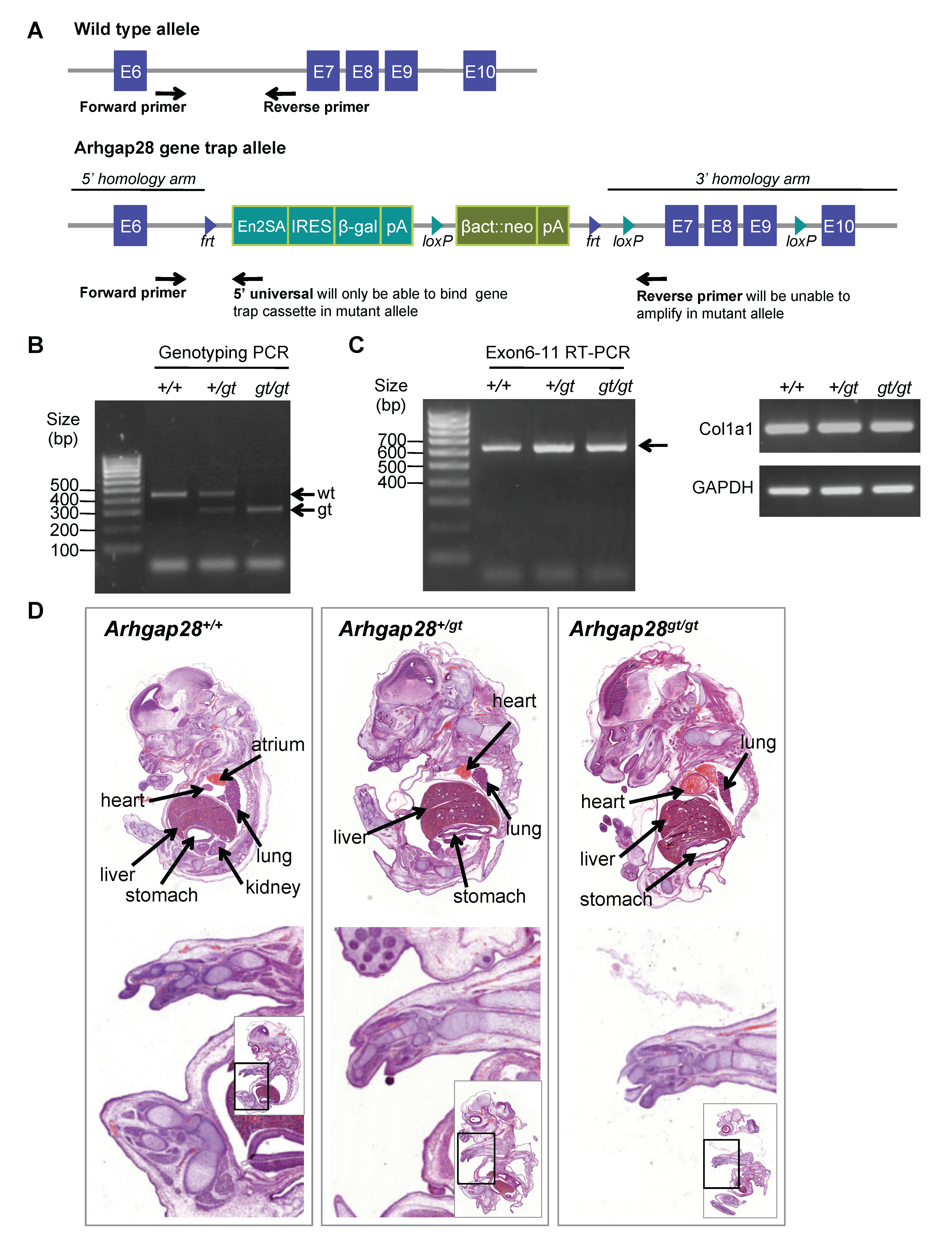

Supplement: Figure S2 — Arhgap28gt mice are normal and express Arhgap28 due to unsuccessful gene trapping. A. Schematic showing the genotyping strategy for identifying the presence of the gene trap cassette targeted to the Arhgap28 gene. B. DNA was isolated from wild type, Arhgap28+/gt and Arhgap28gt/gt mutant neonatal tail tendons cells to confirm genotypes. DNA from wild type (+/+) animals will only produce a 493 bp product whereas DNA from homozygous (gt/gt) animals will only produce a 354 bp product and DNA from heterozygous (+/gt) animals will produce both bands. C. RNA was also isolated and RT-PCR was performed to detect the expression of Arhgap28 transcript spanning from exon 6 to 11, Col1a1 and Gapdh. D. Sagittal sections of wild type (+/+), heterozygous (+/gt) and homozygous Arhgap28gtmutant (gt/gt) embryos at gestation day E15.5 stained with H&E and close-up of the limbs. (TIFF) [file pone.0107036.s002.tiff]

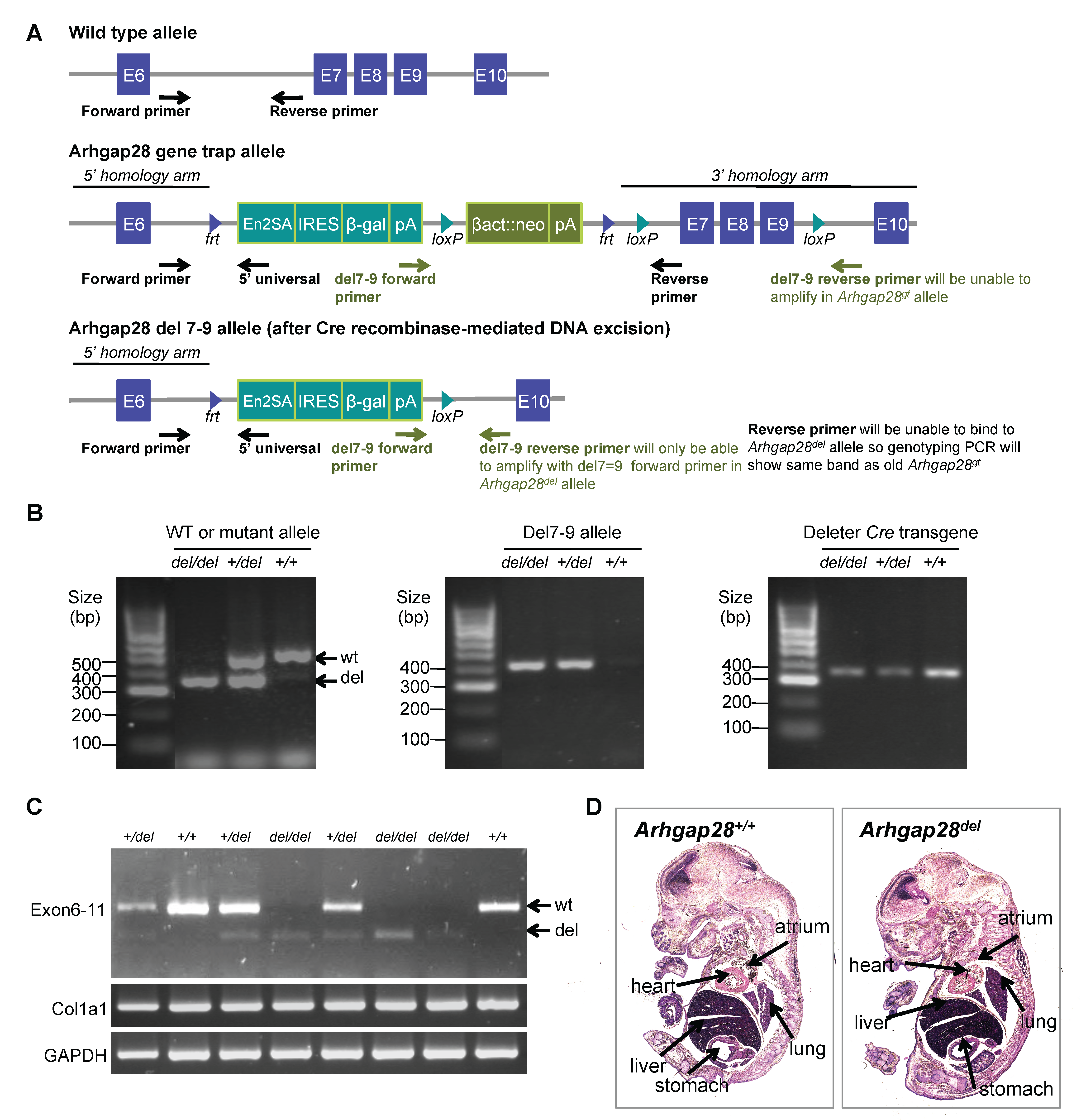

Supplement: Figure S3 — Arhgap28del mice express an Arhgap28 transcript lacking exons 7 to 9. A. Schematic showing the genotyping strategy for identifying the presence of the gene trap cassette targeted to the Arhgap28 gene and for the detection of Arhgap28 del7-9 KO allele. B. Representative gel image of genotyping PCR products. In the first genotyping PCR which distinguishes between wild type (493 bp) or mutant Arhgap28 allele (either the Arhgap28gt or Arhgap28del allele; 354 bp). The second genotyping PCR tests for the presence of the Arhgap28del allele, the product of the mutant Arhgap28 allele after Cre recombinase-mediated DNA excision (400 bp). The third genotyping PCR tests for the presence of the deleter Cre transgene (350 bp). C. RNA was isolated from wild type, Arhgap28+/del and Arhgap28del/del pups and RT-PCR was used to detect expression of wild type Arhgap28 (634 bp) and Arhgap28del (338 bp) transcripts spanning from exons 6 to 11. RT-PCRs for Col1a1 and Gapdh was used as loading controls. D. Sagittal sections of wild type and homozygous Arhgap28del embryos at gestation day E15.5 stained with H&E. (TIFF) [file pone.0107036.s003.tiff]
