# Supplementary material for: Arhgap28 Is a RhoGAP that Inactivates RhoA and Downregulates Stress Fibers
Source: PLoS One. 2014 Sep 11;9(9):e107036. doi: 10.1371/journal.pone.0107036 (PMC4161385; doi:10.1371/journal.pone.0107036)
Supplement: Table S1 — Summary of studies in which Arhgap28 is identified as a candidate of interest. (DOCX) [file pone.0107036.s006.docx]

| **Reference** | **Tissue (and organism if not human)** | **Analysis and observations** |
| --- | --- | --- |
| Zhao *et al.*, 2007 | stromal cell cultured from prostate tissue | cDNA microarray; Arhgap28 was upregulated in stromal cell cultures derived from benign prostatic hyperplasia compared to cells derived from cancerous prostate tissue |
| Anraku *et al.*,2008 | lung tissue used for transplantation prior implantation | cDNA microarray; Arhgap28 was upregulated in tissues from patients who died as a consequence of primary graft dysfunction compared to lung tissues from case-matched patients who had excellent outcomes |
| Schilling *et al.*, 2008 | osteoblasts and adipocytes derived from the differentiated hMSC (bone marrow) | cDNA microarray; Arhgap28 was upregulated 24 hours after initiating the trans-differentiation of adipocytes into osteoblasts and 24 hours after initiating the trans-differentiation of osteoblasts into adipocytes |
| Fèvre-Montagne *et al.*, 2009 | meningioma tissue | cDNA microarray; Arhgap28 was upregulated in atypical meningiomas and anaplastic meingiomas compared to low-grade meningiomas |
| Floyd *et al.*, 2009 | atherosclerotic plaque tissue from aorta of *ApoE^-/-^* mice | cDNA microarray; Arhgap28 was upregulated in plaques of mice exposed to concentrated ambient air particles compared to mice exposed to filtered air |
| Wetmore *et al.*, 2010 | serum mRNAs from rat liver | cDNA microarray; Arhgap28 was upregulated in serum after treatment with hepatotoxins, acetaminophen but not after treatment with D-(+)-galactos-amine |
| Hashimoto *et al.*, 2011 | prefrontal cortex of mice | cDNA microarray; Arhgap28 was upregulated in male withdrawal seizure-resistant mice compared to female mice and to male and female withdrawal seizure-prone mice |
| Lippi *et al*., 2011 | primary culture of murine hippocampal neurons | siRNA transfection; knockdown of Arhgap28 mRNA did not significantly alter spine morphology (filopodia or neuronal spines) |
| Wansbury  *et al.*, 2011 | mammary primordium of E12.5 mouse embryos | cDNA microarray; Arhgap28 was upregulated in the mammary mesenchyme compared to the mammary primordial bud epithelium |
| Chan *et al*., 2012 | spermatozoa of rat model for human testicular cancer treatment | DNA methylation analysis; Arhgap28 gene was hyper-methylated in spermatozoa of rats after treatment with bleomycin, etoposide and *cis*-platinum |
| Gewurz *et al.*, 2012 | HEK cells containing GFP reporter for NFκB activation | siRNA screen; knockdown of Arhgap28 reduced Epstein Barr virus latent membrane protein mutant- and interleukin-1β-stimulated but not TNFα-stimulated, NFκB activation |
| Hadj-Hamou *et al.*, 2012 | breast tissue angiosarcomas | cDNA microarray; Arhgap28 was downregulated in radiation-induced tumours compared to primary tumours |
| Paramanik and Thakur, 2012 | mitochondrial extract of mouse brain tissue | pull-down assay and motif scanning; Arhgap28 was found to interact with ligand-binding domain of oestrogen receptorβ and seven consensus motif for casein kinase 2 phosphorylation sites |
| Pradat *et al.*, 2012 | deltoid muscle | cDNA microarray; Arhgap28 was upregulated in patients with advanced amyotrophic lateral sclerosis compared to controls and patients with early state of amyotrophic lateral sclerosis |
| Yang *et al.*, 2012 | CD133^+^ cells cultured from glioblastoma multiforme (brain tumour) | cDNA microarray; Arhgap28 was upregulated in these radio-resistant tumour initiating (CD133^+^) cells after being cultured with cancer-preventative polyphenol resveratrol |
| Zhu *et al.*, 2012 | embryonic palatal mesenchyme cells | cDNA microarray; Arhgap28 was downregulated in cells treated with TGFβ |

**Table S1. Summary of studies in which *Arhgap28* is identified as a candidate of interest.**
