# Supplementary material for: Arhgap28 Is a RhoGAP that Inactivates RhoA and Downregulates Stress Fibers
Source: PLoS One. 2014 Sep 11;9(9):e107036. doi: 10.1371/journal.pone.0107036 (PMC4161385; doi:10.1371/journal.pone.0107036)
Supplement: Table S2 — PCR primers. (DOCX) [file pone.0107036.s007.docx]

| **Purpose** | **Forward primer 5’-3’** | **Reverse primer 5’-3’** | **Product size** | |
| --- | --- | --- | --- | --- |
| *Arhgap6* (mouse) qPCR | GCTGCTGTCAACATCTGGAA | TCAGGCTCCAACAGGAGAGT | 247 bp | |
| *Arhgap6* (chick) qPCR | ACGATCTTTGGCCCTAACCTGT | ACAACATCTGGGTCGGTCTCTA | 206 bp | |
| *Arhgap18* (mouse) qPCR | GCCCAGACACTCAAGAAAGG | TGTCGTCTACACTGGCTTGG | 153 bp | |
| *Arhgap18* (chick) qPCR | GGCTTATGATCGGGAAAAGCAC | GCAGCGTTCCCCAATATTTCCT | 247 bp | |
| *Arhgap28* (mouse) qPCR | GCCATTCAGCTCAACAGTCA | TAGGGCTTGATGACCCATTC | 212 bp | |
| *Arhgap28* (chick) qPCR | TGGACAAAGAGCCAGAGGTT | GTTTTCTCGTCTCCGCACTC | 216 bp | |
| *Arhgap28* (Exon6-11) RT-PCR | TGCTGTTCACACCAATGGAT | AACATGACCGCAGCTTCTCT | 624 or 388 bp | |
| *Arhgap28-V5-His* RT-PCR | CAAAGATGTCCTGGCGAAAT | AACTAGAAGGCACAGTCGAGG | 233 bp | |
| *DLC1* qPCR | TGTGCTGAGAGAACGTCACC | ACTCCACAGACAGGGACACC | 200 bp | |
| *GAPDH* qPCR | AACTTTGGCATTGTGGAAGG | ACACATTGGGGGTAGGAACA | 223 bp | |
| *COL1A1* RT-PCR | GCCTGCTTCGTGTAAACTCC | TTGGTTTTTGGTCACGTTCA | 224 bp | |
| *RhoA* qPCR | GTGGATGGGAAGCAGGTAGA | TTGTTCCCAACCAGGATGAT | 212 bp | |
| *Rac1* qPCR | CTGAAGTGCGACACCACTGT | CTTGAGTCCTCGCTGTGTGA | 203 bp | |
| *Cdc42* qPCR | CTGTTTCCGAAATGCAGACA | AATCCTCTTGCCCTGCAGTA | 201 bp | |
| *RhoQ* qPCR | CTCATGAGCTATGCCAACGA | GGATTTACCACGGAGAAGCA | 203 bp | |
|  |  |  | **larger isoform** | **smaller isoform** |
| Overlapping PCR #1 | CGTTGGTCTCGGTCTTTGTT | CTCTGTAGGGCCACAAAACC | 780 bp | 780 bp |
| Overlapping PCR #2 | CCTGCGGGAAATTGAGAGTA | GTGGCGGATTTTCTTCATGT | 808 bp | 808 bp |
| Overlapping PCR #3 | GAAGAACGTTCCAGCAGAGG | GTTCATGCGGTTCTTGGATT | 766 bp | 766 bp |
| Overlapping PCR #4 | AGAGAAGCTGCGGTCATGTT | TAGGGCTTGATGACCCATTC | 821 bp | - |
| Overlapping PCR #5 | AGAGAAGCTGCGGTCATGTT | CAGGGATACCTGCCTGTGAT | - | 821 bp |
| Overlapping PCR #6 | GCCATTCAGCTCAACAGTCA | AGGAGAAACGGAAGACACCA | 800 bp | - |
| Overlapping PCR #7 | CATGGTGTGAGGGAGCTTTC | GGCTCCACAGGTAAGGAACA | 759 bp | - |
| Overlapping PCR #8 | CAAAGATGTCCTGGCGAAAT | TCTCTTTTGTGGGCAAATCC | - | 813 bp |
| Overlapping PCR #9 | GCTTGTGGAAGCTGGAGACT | CAGGGATACCTGCCTGTGAT | 767 bp | - |
| Overlapping PCR #10 | GGGTTTCTGTGTGTCCCTGT | TCTCTTTTGTGGGCAAATCC | 752 bp | - |
| Overlapping PCR #11 | TCGGCTATGCCTTTTTGTTC | AAATCCATGGAGAGCAGTGG | 826 bp | 826 bp |
| Overlapping PCR #12 | TGCCGTTAGCTTTCAGTGTG | GGAAAATAGTTGAAAATGCCTGTC | 443 bp | 400 bp |
|  |  |  | **wild type** | ***Del*** |
| Overlapping PCR #13 | TGCTGTTCACACCAATGGAT | AACATGACCGCAGCTTCTCT | 624 bp | 388 bp |
| Overlapping PCR #14 | GAAGAACGTTCCAGCAGAGG | GTTCATGCGGTTCTTGGATT | 766 bp | - |
| Genotyping *Arhgap28* wild type, *Arhgap28^gt^* and/or *Arhgap28^del^* alleles | CCCGAATACCTAGCAGTGGA | Wild type allele TACCGAGATCTGGGGAACAG Mutant allele CAACGGGTTCTTCTGTTAGTCC | 493 bp or 354 bp, respectively | |
| Genotyping *Arhgap28^del^* allele | CCCCCTGAACCTGAAACATA | GAGGCAGGAGGATCTCTGTG | 400 bp | |
| Genotyping *Cre* transgene | TTCAATTTACTGAACGTACA | AAACAGCATTGCTGTCACTT | 350 bp | |

**Table S2. PCR primers.**
